# Cost‐effectiveness analysis of antiviral treatment in the management of seasonal influenza A: point‐of‐care rapid test versus clinical judgment

**DOI:** 10.1111/irv.12359

**Published:** 2016-01-29

**Authors:** Léon Nshimyumukiza, Xavier Douville, Diane Fournier, Julie Duplantie, Rana K. Daher, Isabelle Charlebois, Jean Longtin, Jesse Papenburg, Maryse Guay, Maurice Boissinot, Michel G. Bergeron, Denis Boudreau, Christian Gagné, François Rousseau, Daniel Reinharz

**Affiliations:** ^1^ Faculté de MédecineDépartement de médecine sociale et préventiveUniversité LavalQuébecQCCanada; ^2^ Faculté des sciences et génieDépartement de génie électriqueUniversité LavalQuébecQCCanada; ^3^ Centre de recherche en infectiologie (CRI)CHU de Québec (CHUQ)QuébecQCCanada; ^4^ Faculté de médecineDépartement de microbiologie‐infectiologie et d'immunologieUniversité LavalQuébecQCCanada; ^5^ Faculté de MédecineDépartement de pédiatrieUniversité McGillMontréalQCCanada; ^6^ Faculté de médecineDépartement des sciences de la santé communautaireUniversité de SherbrookeLongueuilQCCanada; ^7^ Faculté de sciences et de génieDépartement de physique, génie physique et d'optiqueUniversité LavalQuébecQCCanada; ^8^ Faculté de sciences et de génieDépartement de chimieUniversité LavalQuébecQCCanada; ^9^ Faculté de médecineDépartement de biologie moléculaire, biochimie médicale et pathologieUniversité LavalQuébecQCCanada; ^10^ Unité de recherche en génétique humaine et moléculaireAxe Santé des populations et pratiques optimales en santéCentre de recherche du CHU de Québec (CHUQ)QuébecQCCanada

**Keywords:** Antiviral treatment, cost effectiveness, point‐of‐care rapid test, seasonal influenza, simulation

## Abstract

**Background:**

A point‐of‐care rapid test (POCRT) may help early and targeted use of antiviral drugs for the management of influenza A infection.

**Objective:**

(i) To determine whether antiviral treatment based on a POCRT for influenza A is cost‐effective and, (ii) to determine the thresholds of key test parameters (sensitivity, specificity and cost) at which a POCRT based‐strategy appears to be cost effective.

**Methods:**

An hybrid « susceptible, infected, recovered (SIR) » compartmental transmission and Markov decision analytic model was used to simulate the cost‐effectiveness of antiviral treatment based on a POCRT for influenza A in the social perspective. Data input parameters used were retrieved from peer‐review published studies and government databases. The outcome considered was the incremental cost per life‐year saved for one seasonal influenza season.

**Results:**

In the base‐case analysis, the antiviral treatment based on POCRT saves 2 lives/100 000 person‐years and costs $7600 less than the empirical antiviral treatment based on clinical judgment alone, which demonstrates that the POCRT‐based strategy is dominant. In one and two way‐sensitivity analyses, results were sensitive to the POCRT accuracy and cost, to the vaccination coverage as well as to the prevalence of influenza A. In probabilistic sensitivity analyses, the POCRT strategy is cost‐effective in 66% of cases, for a commonly accepted threshold of $50 000 per life‐year saved.

**Conclusion:**

The influenza antiviral treatment based on POCRT could be cost‐effective in specific conditions of performance, price and disease prevalence.

## Introduction

Influenza causes over than 4000 deaths in Canada annually, the large majority of which are attributable to type A strains.^1^ While vaccination is the cornerstone of prevention, antiviral drugs are the only specific medication for influenza infection. They are most effective at reducing complications when used early (within 48 hour of illness onset).^2^ Management of influenza infections remains a challenge, mainly because of the difficulty in making a rapid and accurate diagnosis. Clinical diagnostic criteria lack accuracy compared with laboratory methods because influenza causes a wide spectrum of disease that is often clinically indistinguishable from other respiratory infections.^3^ However, results of traditional microbiological tests are not available to practitioners in a clinically relevant timeframe, obliging clinicians to use an empirical approach when suspecting influenza A infection. Furthermore, currently available rapid diagnostic tests for influenza A have a low sensitivity.^4^ Thus, there is some interest in the development of new diagnostic tools that are simple enough to be used at the bedside (i.e., point‐of‐care [POC]) for rapid and reliable diagnosis. Such rapid tests would allow an early and targeted use of antiviral drugs for patients with influenza infection, thus improving their outcomes.

Economic studies comparing rapid testing to clinical diagnosis of influenza remain inconclusive. Indeed, some studies suggested that, in most cases, clinical judgment combined with antiviral treatment is the most cost‐effective strategy,^5^ while others suggested that testing may be the most cost‐effective strategy.^6^ In addition, even if studies agreed that the cost‐effectiveness of rapid tests is sensitive to their accuracy and costs, to the prevalence of influenza and its complications as well as to the vaccination status, no one has, to our knowledge, specifically estimated the thresholds of accuracy and costs from which the rapid testing strategy could be considered as cost‐effective compared to clinical judgment.

Considering that developing such a test is time‐ and resource‐consuming, it is therefore relevant to define the thresholds of accuracy and costs from which a new rapid POC test is expected to be cost‐effective and to do it before its development and its implementation in general practice.

Using a hybrid transmission and decision analytic economic model, the objective of this study was to address two principal questions: (i) Is an antiviral treatment based on a rapid POC test for influenza A cost‐effective compared to the empirical treatment based on clinical judgment? and (ii) At what thresholds of POC test accuracy (sensitivity and specificity) and cost is the testing strategy cost‐effective?

We aimed to provide generic information from an economic point of view regarding the optimal characteristics of POC tests (accuracy and cost) in order to help industries to decide about the interest of developing such assays, and to help decision‐makers to establish the relevance of implementing such assays within a healthcare system.

## Methodology

### Model structure

**Figure 1:**
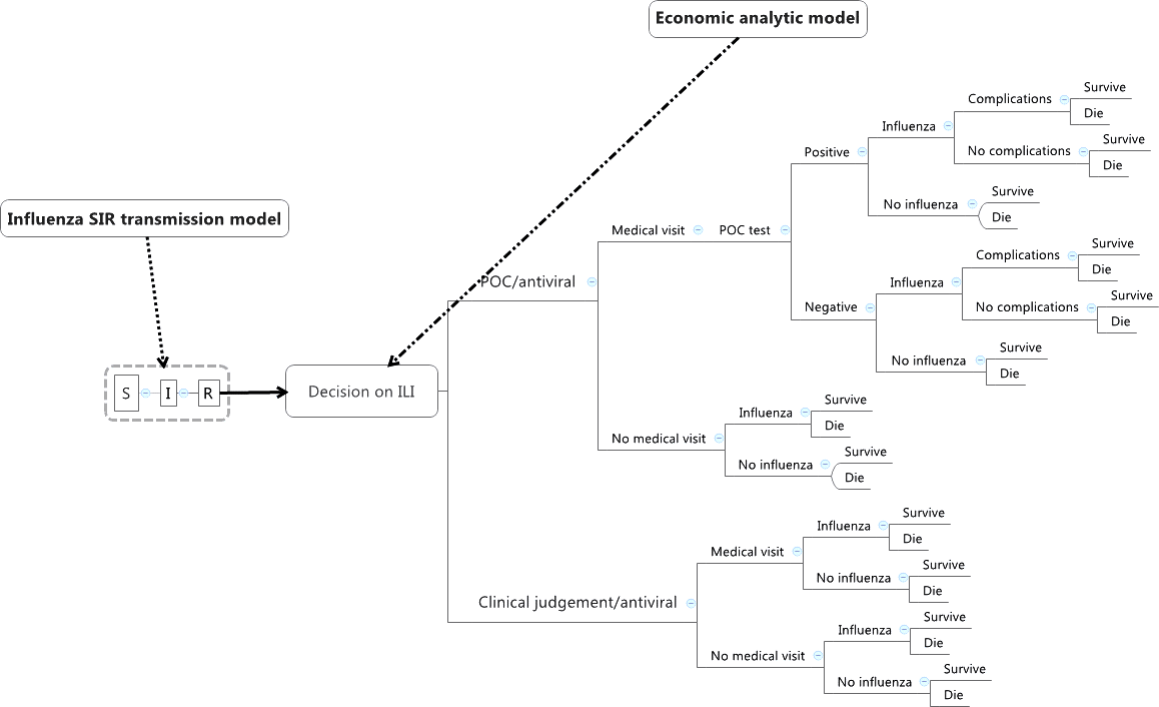
Influenza analytic decision model.

**Figure A1:**
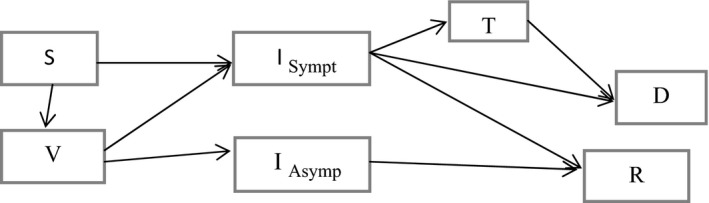
Schematic diagram of infection progression and intervention pathways. S = susceptible, I_symp_ = infected and symptomatic, I_Asymp_ = infected and asymptomatic, T = under treatment, R = recovered, V = vaccinated, D = death.

Using SCHNAPS,^7^ an agent‐level Markov model (SPLMM)‐based simulator running on the network of supercomputers of the CLUMEQ consortium (www.clumeq.ca), an hybrid transmission and decision analytic economic model (Figure 1 and Figure A1 of Appendix 1) was built to simulate the expected economic performance of a potential rapid test (POC) for the diagnosis and early appropriate antiviral treatment of seasonal influenza A compared to clinical judgment. The model aimed at being representative of the population of Quebec (Canada). We performed the study under the societal perspective. Results were produced for a one‐year influenza season. The transmission model is based on a standard SIR (susceptible, infected, and recovered) compartmental model that can be described by three differential equations:

dS/dt=−βIS

dI/dt=βSI−γI

dR/dt=γI

where S = susceptible, I = infected, R = recovered, β = Infectious contact rate, γ = recovery rate, and 1/γ = infectious period. This model considers cycles of one day each and assumes that if a person is infected, he becomes infectious for a time, and that once he has recovered, he becomes immune for the rest of that influenza season. The total population size at time *t* is given by N (*t*) = S (*t*) + I (*t*) + R (*t*). We assumed a homogenous mixing in the population which means that each individual has the same probability of having a contact with any other individual in the population. Transmission probabilities given a contact were modeled in such a way that the influenza basic reproductive number *R*_0_ of seasonal influenza was equal to what was found in the published literature.^8^

The model assumes that individuals who remain asymptomatic will not seek medical care. It considers that a fraction of symptomatic influenza‐like illness cases, those who do not feel very sick, will not consult a physician. It assumes also that half of the symptomatic patients who did not seek medical care would be self‐treated with over‐the‐counter medications. We considered that the remaining symptomatic cases would seek medical help in an outpatient clinic or in an emergency department.^9^ We assumed that all patients considered as influenza positive and seen in the first 48 hours after the beginning of symptoms will get oseltamivir antiviral treatment two times a day for five days, with, as a consequence, a reduction in the duration of the influenza illness and the probabilities of influenza complications like pneumonia and death.

The model considers the loss of productivity related to absence from work.^10^ We assumed that children <12 years old would require the presence of one adult caregiver when sick and that an adult would need to take leave from work. We assumed that the duration of absence from work of individuals who were hospitalized was equivalent to the hospitalization length of stay according to the Quebec diagnosis‐related group (DRG) database.

Input parameters were retrieved from an extensive literature search and peer‐reviewed published studies prioritized, for the choice of the baseline parameters, according to the type of study (randomized control, meta‐analyses, observational, economic modeling) in the following setting order: Quebec, other provinces of Canada, United States of America (USA), Europe, and Australia.

Outcomes were the total costs and influenza‐related deaths. The primary outcome for C/E analysis was the incremental cost per life‐year saved (IC/LYS).

### Population

We stratified the virtual population into three age groups: <19 years; 20–64 years, and >65 years, and considered for each group its vaccination status. Transmission between these age groups was based on data on social contacts and mixing patterns from POLYMOD survey conducted in the European Union.^11^ We considered that 90% of all vaccinated cases were vaccinated in November, the month in which vaccination campaigns against influenza are organized in the province of Quebec. Age group‐specific vaccination data were derived from Quebec database. The vaccine efficacy was modeled according to age.^12^ As we considered only one influenza season, the population size was assumed to be constant for the duration of this flu season. In the base case scenario, we assumed that 10% of the population had an acquired immunity against influenza and could not be infected.

### Model scenarios

We considered two scenarios, namely (i) empirical antiviral treatment based on clinical judgement and (ii) antiviral treatment guided by a point‐of‐care rapid test (POCRT). We assumed also that antiviral treatment would be prescribed as a treatment not as prophylaxis.

### Costs

Costs were estimated in Canadian dollars for the fiscal year 2011–2012 (1 Canadian dollars = 1 US dollar) which was used to calculate unit prices which were provincial averages calculated from the Quebec government databases. These costs have taken into account of direct healthcare costs and indirect costs related to loss of productivity caused by workplace absenteeism. Healthcare cost items included influenza‐related diagnosis and treatment, influenza‐related hospitalizations, diagnosis and management of post‐influenza complications, vaccination campaigns, and patient average out pocket of over‐the‐counter medications. Unit prices of clinical activity centers were increased to reflect support activity centers. Costs for laboratory and imaging tests were based on the technical units in the province of Quebec. The average cost of national campaigns of influenza vaccination in Quebec was obtained from a Quebec National Institute of Public Health survey on costs and efficacy of the Quebec influenza vaccination program. Doctor costs were retrieved from the healthcare fees paid by the Quebec public insurance to physicians (general practitioners and specialists). The cheapest drug on the list of products covered by the public healthcare insurance was used to which 6% was added for wholesalers and the pharmacist's prescribing fee paid by the public insurance. For antiviral treatment, we considered only oseltamivir as it is the most prescribed antiviral product (90%) in Canada. The costs of POC tests were retrieved from published studies and from experts’ opinion. Loss of productivity costs was valued using the human capital method. Values were retrieved from Quebec government database on employment.

### Simulation and process validation

Before starting the simulations, the decision model and input parameters were validated by three clinicians and epidemiologist experts knowledgeable in influenza prevention, infections, diagnosis, and treatment. To produce a distribution curve, simulations for each option were repeated 1000 times, each time on a newly generated (*i.e.,* different) virtual population. Then, data produced were validated by comparison with expected data (such as the number of influenza hospitalization cases, excess mortality rates per age, costs, and effectiveness of interventions) to ensure the validity of the model. For example, our model predicted a death rate of 14·2/100 000 for the current situation which is very close to the rate of 13/100 000 observed in Canada.^1^

#### Sensitivity analyses

We performed sensitivity analyses on key input parameters affecting the cost‐effectiveness of both scenarios. These parameters include, for example, the *R*_0_ value (the basic reproduction number) and the relative effectiveness of vaccination and antiviral treatment, the accuracy, and cost of POC test. One‐way sensitivity analyses were performed to evaluate the eventual impact of each single parameter on the results. We tested the minimum and the maximum (from the 95% confidence intervals) value for each of these variables. Bivariate sensitivity analyses were thereafter performed on the sensitivity and specificity as well as cost of the POC test. Finally, Monte Carlo simulations were used for probabilistic sensitivity analyses in which all parameters were varied concomitantly taking into account their distribution function. We assumed that event probabilities followed a beta distribution that costs followed a gamma distribution while relative risks were assumed to have a lognormal distribution. Finally, the cost‐effectiveness scatterplot and the cost‐effectiveness acceptability curve were produced to better define the probability of being cost‐effective given a ceiling ratio.

## Results

In the base case scenarios for which the cost of a hypothetical point‐of‐care test is set at CAD$ 25 per test, the antiviral treatment guided by POCT appeared as a dominant approach, *that is,* it is more effective and less expensive than the empirical antiviral treatments based on clinical judgment.

Results suggest that, when applied to the population of Quebec (approx. 8 000 000 inhabitants), a rapid POC test would accelerate the diagnosis of influenza and initiate a treatment with antiviral drug more quickly and to more individuals. This would save 154 lives a year and cost $ 605 840 less compare to the empirical antiviral treatment based on clinical judgment (Table 1).

**Table 1:** Model input parameters

Parameter		Base case	Range for sensitivity analysis	Distribution	Source
General population (N)	<19	442 191	NA		Quebec Institute of Statistics
	20–64	401 786			
	>65	40 020			
Population employed (%)		60	55–70	Beta	
*R*_0_		1·2	0·9–2·1	Normal	8
Infectious period		3	2–4	Normal	13
Infection duration		7	5–10	Normal	14
Probability to be initially infected		0·0001	0·00001–0·0005	Gamma	Assumption
Number of contacts (N)		13	3–20	Normal	11
Proportion of symptomatic individuals (%)		67	30–100	Beta	15
Quebec vaccination coverage	<2	25·2	15–35	Beta	Quebec Institute of statistics
	3–49	7·6	5–20		
	>50	41·8	36–70		
Probability of ILI during season (%)	<4	20·3	15–25	Beta	9
	5–17	10·2	8–12		
	18–64	6·6	6–7		
	>65	9	±0·024		
Probability that ARI is Influenza in context of seasonal (%)		77	44–87	Beta	16
Probability that Influenza is type A (%)		66·1	20–90	Beta	17
Probability of infection if vaccinated (%)	<19	36	30–40	Beta	12
	20–69	17·5	15–25		
	>70	35	30–40		
Performance of physician < 48 h (%)	Sensibility	67	39–86	Beta	18
	Specificity	96	81–99		
Performance POC rapid test (%)	Sensitivity	74	67–100	Beta	4
	Specificity	99	98–100		
Probability of previous immunity (%)		10	0–15	Beta	Expert opinion
Probability of medical visit %	<5	48	40–60	Beta	9
	5–17	35	30–40		
	18–64	37	30–40		
	>65	72	65–80		
Probability of medical visit < 48 hours (%)		59	30–70		19
Probability of emergency visit (conditional to medical visit) (%)	< 5	26	20–35	Beta	9
	5–64	20	10–30		
	>65	57	45–80		
Probability of hospitalization (%)	< 5	1·41	0·7–2·1		
	5–64	1·02	0·5–1·6		
	>65	4·21	3–6		
Probability of death (conditional to hospitalization) (%)	< 5	0·4	0·2–0·6	Beta	
	5–49	2	0–4		
	50–64	7	3–10		
	>65	16	10–22		
Probability of pneumonia influenza related	< 5	2·4	1·5–5	Beta	20
	5–17	1·18	0·5–3		
	>18	1·5	0·5–4		
Probability self‐medicated (%)		50·9	10–60	Beta	Quebec Institute of Statistics and national Institute of public health
Number of days work‐off		2	1–4	Normal	10
Efficacy of antiviral treatment	Relative risk on mortality	0·21	0·06–0·80	Lognormal	21
	Relative risk on hospitalization for adults	0·92	0·57–1·50	Lognormal	22
	Relative risk on pneumonia	0·55	0·22–0·90	lognormal	
	Reduction of length of influenza	24 hour	0–48 hour	Normal	16
	Relative risk on antibiotic's use	0·33	0·29–0·48	Lognormal	23
Costs (CAD$)					
Outpatient department visit	≤70 years	149·64		Fixed	Quebec's government databases
	>70 years	151·34			
Emergency department visit	≤70 years	413·195			
	>70 years	416·245			
Hospitalization (4 days) + inpatient visits		7460	3600–11 000	Gamma	
Pneumonia	≤70 years	450·3	200–3000	Gamma	
	>70 years	485·6	200–3000		
Oseltamivir (Tamiflu^®^)		30 (adults); 15 (children)			
Rapid test (POC)		25	5–50	Uniform	5 and expert opinion
Vaccine		20	15–30	Triangular	Quebec National Institute of public health *(INSPQ)*
Workday lost (8 hours/day)		170/day (mean)	100–1000	Gamma	Quebec Institute of Statistics (*ISQ)*

**Table 2:** Base case results

Strategy	Cost/100 000 person‐years	Incremental cost/100 000 person‐years	Deaths/100 000 person‐years	Life‐year saved/100 000	Cost/Life‐year saved
POC test and antiviral	2 982 574		12·35		
Clinical judgment and antiviral	2 990 147	7573	14·27	1·92	Dominated

**Figure 2:**
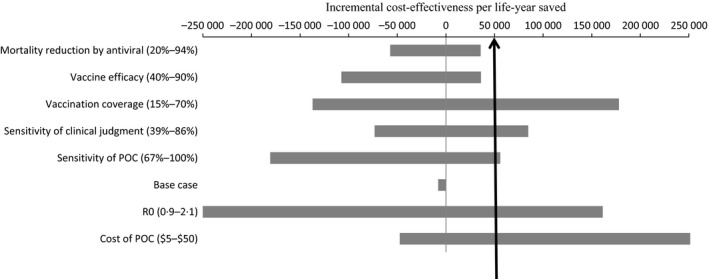
Tornado diagram presenting results of univariate sensitivity analyses. The horizontal axis show various Incremental cost‐effectiveness per life‐year saved. At a threshold of $ 50 000 per life‐year saved, the POC strategy remain robust only for two parameters: the efficacy of antiviral in reduction of mortality and the vaccine efficacy.

The univariate sensitivity analyses show that our results were robust (i.e., the POCT strategy remains the most cost‐effective) to the antiviral effect on mortality and to the efficacy of vaccine against influenza. On the other side, the results were sensitive to the basic reproductive number (*R*_0_), to the sensitivity and the cost of POCT, to the performance of physician, and to vaccination coverage. These results are presented in Figure 2.

Regarding the basic reproduction number (*R*_0_), results show that when it is set at 0·9, the POCT strategy is not cost‐effective, while it is very dominant when the *R*_0_ is set at 2·1. The same conclusion applies to the sensitivity of POCT. However, when sensitivity of clinical judgment, vaccination coverage, and cost of POC are set to their minima values, the POCT strategy remains dominant, whereas it loses its cost‐effectiveness if the parameters are set at their maxima values if the cost‐effectiveness threshold is fixed at $50 000 per life‐year saved.

**Figure 3:**
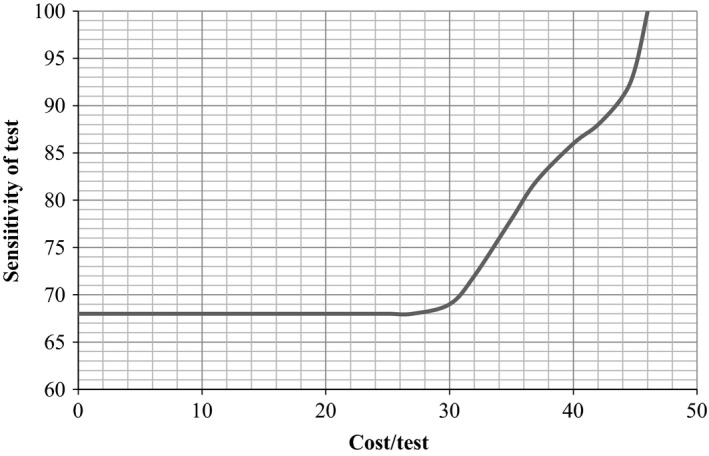
Two‐way sensitivity analyses by sensitivity and cost/per POC test.

The two‐way sensitivity analyses on the sensitivity and the cost of POC test showed that the POCT strategy is cost‐effective if the cost of POC is less than $32 and if its sensitivity is above 68%. However, when the test exceeds 46$ per test, the POCT strategy is not cost‐effective for a threshold of $ 50 000 per life‐year saved even if the sensitivity of the POC test is 100%. These results are presented in Figure 3.

**Figure 4:**
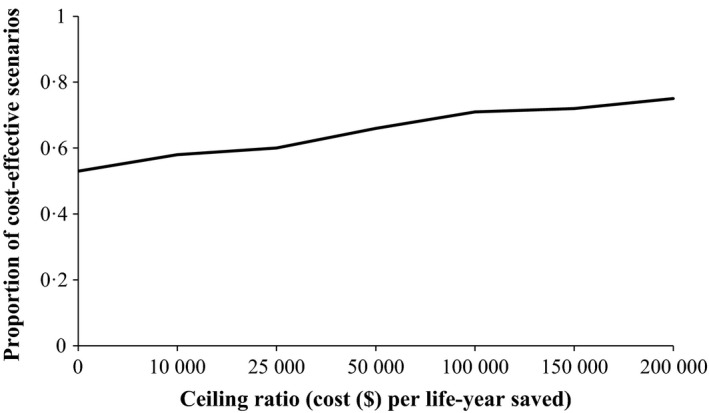
Probabability sensitivity analysis results: cost‐effectiveness acceptability curve: antiviral based on POCT versus empirical antiviral treatment.

The results of probability sensitivity analyzes are presented in Figure 4 which shows that the antiviral treatment guided by POCT is the most cost‐effective option compared to the empirical antiviral treatment guided by clinical judgment in 66% of simulations if the threshold ceiling ratio (cost/life‐year saved) is set at $ 50 000.

## Discussion

The present study had two main objectives: (i) to determine whether antiviral treatment based on a rapid POCT for influenza A is cost‐effective compared to the empirical antiviral treatment based on clinical judgment and (ii) to determine the thresholds of key test parameters (sensitivity, specificity, and cost) at which the POCT strategy appears to be cost‐effective.

Considering the baseline values of sensitivity, specificity, and cost to be 74%, 99%, and $25, respectively, for a POC test; the antiviral treatment based on this test appears dominant as compared to empirical antiviral treatment based on clinical judgment. One‐way sensitivity analyses show that the results remain robust for only two parameters (antiviral efficacy on mortality and vaccine efficacy for influenza): POCT strategy is dominant if high values for these parameters are considered and is cost‐effective at a threshold of $50 000 per life‐year saved if low values are considered. However, results were not robust to one‐way sensitivity analyses when other parameters were varied: The POCT strategy option is either dominant or not cost‐effective when the cost‐effectiveness threshold is set at $50 000 per life‐year saved. In two‐way sensitivity analyses, the antiviral treatment based on POCT is not cost‐effective if sensitivity is less than 68% and if cost exceeds $46 per test. In probabilistic sensitivity analyses, the POCT strategy is cost‐effective in 66% of cases, when a threshold of $50 000 per life‐year saved is fixed.

Our findings are compatible with those of Nagase *et al*. 6 who showed that oseltamivir treatment based on POC test is a dominant option compared to conventional approaches without screening test in the baseline scenario and could be cost‐effective in 80% of cases according to the cost‐effectiveness acceptability curve produced by Monte Carlo simulations. Nagase *et al*. determined that the sensitivity of the POC test must be higher than 90% in the non‐epidemic periods or higher than 60% in epidemic periods for the screening test to be cost‐effective.

What can we learn from this exercise? Our study was able to identify conditions that could influence the potential economic impact of a hypothetical rapid test (POC) for the detection of seasonal influenza. Such conditions included the cost and the accuracy of the POC test, the performance of physicians’ diagnosis and management abilities in detecting influenza cases, the population vaccination coverage, and the influenza basic reproduction rate. It seems therefore important to analyze these conditions in order to better determine the interest of such a new POC technology. Computer simulations are thus highly suited for handling these numerous factors that must be taken into account.^13^

This study has some limitations. The first limitation is the complexity of mapping the reality in simulation models. Some simplifications and assumptions were inevitable in the modeling approach. For example, in our SIR model, we considered that all individuals had the same influenza transmission probability given a contact. This may not fully reflect the complexity of influenza transmission dynamics. Moreover, we did not take into account the side effects of the antiviral treatment. However, common side effects of oseltamivir are mild and self‐limited, whereas more serious side effects are rare; neither would have strongly influenced our conclusions.

The second limitation is related to the input data parameters used which were, in majority, retrieved from retrospective observational studies which comprise inherent uncertainties due to potential biases related to their design. However, our extensive sensitivity analyses allowed us to identify influential factors and thus describe the scenarios in which our results would be valid.

Finally, our model is limited by the consideration of a single perspective, *that is,* the public healthcare perspective. The addition of the patients’ perspective could increase the incremental cost‐effectiveness ratio (ICER) especially in the case of the clinical judgment option where influenza complications are high compared to the POC test option; these complications would certainly incur expenses for patients.

Despite these limitations, this study suggests that the antiviral treatment based on POC test could be cost‐effective if conditions that influence the economic impact of such POC test for the detection of seasonal influenza A are well evaluated. Computer simulations are highly suited for handling these numerous factors that have to be taken into account. With simulations, it is possible to estimate before the technology is developed, the threshold values of the parameters directly related to this test (sensitivity, specificity, and cost) for which the technology could become economically valuable. Computational simulations could thus inform the decisions of researchers and industry during the development of a new technology to stay within the parameters that would make the product cost‐effective. However, it is very important to consider the health system setting on which we base our estimates. Indeed, it should be noted that our findings were based on the Canadian context (a quasi‐exclusive public healthcare system). Thus, confirmation in other healthcare jurisdictions is needed, especially in private‐based health systems where costs of care are relatively high or in developing countries where the cost of POC could be an obstacle.

## Author contributions

FR, MGB, NL, RD, DR, JD, JL, JP, MG, and IC involved in conception, design, acquisition, and validation of data; DF, XD, and CG performed computer simulations; NL, DR, and FR performed analysis and interpretation of results; DR and NL drafted the article; DR, FR, JP, JL, MG, MB, and DB critically revised the article; and all authors approved the final version of article.

## Disclosure/competing interest's declaration

MGB discloses income, grant funding and the ownership of stocks from GenePOC. DB received funding from GenePOC.

## Transparency declaration

All authors declare that the manuscript is an honest, accurate, and transparent. No important aspects of the study have been omitted.

## Appendix 1

### Simulator, model description and input parameters.

#### SCHNAPS

SCHNAPS is a generic simulator designed for healthcare modelling and simulations, parameterizable by configuration files and usable by non‐programmers such as public health specialists. SCHNAPS stands for SynCHroNous Agent‐ and Population‐based Simulator. Before starting to use the GUI, a small knowledge of how SCHNAPS behaves when running simulations is required. Mainly, two different steps occur during simulations (individual virtual generation and simulation).

At first, SCHNAPS generates virtual individuals. Each individual has his own variables. When supplying a configuration file to SCHNAPS, one has to ensure that these variables are correctly named, initialized, and that the information on how the variables are distributed in the virtual population is correct. Such subjects will be later discussed.

Individuals are generated at the start of a simulation. However, the user can specify additional generations during the simulation.

Once individuals have been generated, the simulator makes sure that each individual evolves during the simulation. Such evolution is done by “routing” the individuals in trees that have been previously created by the user. Probabilities, either variables or fixed in the tree, affect the path taken by the virtual individuals. A path is often configured to modify variables, hence causing the so‐called evolution. The simulation infrastructure is composed of open source tools that are freely available on the Web at http://schnaps.googlecode.com (SCHNAPS) and http://sourceforge.net/projects/lsdsimulatorinp (input GUI).

#### Model description

The model consists of two parts: the stochastic transmission model and economic analytic model. For the transmission model, three basic compartments described by three differential equations were used:

dS/dt=−βIS

dI/dt=βSI−γI

dR/dt=γI

where: S (susceptible), I (infected), and R (recovered).

The total population size at time *t* is given by N (*t*) = S (*t*) + I (*t*) + R (*t*).

The Figure A1 represents the schematic diagram of infection progression and pathway in the health system.

The simulation of this process consists of the following:

1. Interpreting the next event to occur: In the SIR model, an event is defined as a susceptible becoming infected ((S, I, R) (S−1, I + 1, Z)) or an infected recovering or removed from the population ((S, I, R) (S, I−1, R + 1)). The probability of a susceptible person becoming infected is as follows: βS (I/(S + I))/(Iγ + βS(I/(S + I))), and the probability of a removal/recovery is as follows: γ I /(γ I + βS(I/(S + I)).
2. Interpreting the distribution of the time to the next event: The uniform random number generator implemented in SCHNAPS was used. Approximation of both the time to the next event, according to the distribution of the time to the next event, and the transition among states was done, through a Monte Carlo probabilistic structure.

Each individual was explicitly represented in the simulation and was assigned a status: age and vaccination. We assumed a homogenous mixing in the population which means that each individual has the same probability of having a contact with any other individual in the population.

Transmission probabilities given a contact were modeled in such a way that the influenza basic reproductive number *R*_0_ of seasonal influenza was equal to what was found in the published literature. The basic reproductive number (*R*_0_) is the number of infected cases produced by one infective individual in a totally susceptible population, during his/her infectious time and in the absence of any interventions.

The probability of becoming infected depends on four major factors: susceptibility of susceptible individual (vaccination status), number of contacts with infected individuals, infectivity of infected individuals, and duration of the contacts. We considered 1/100 000 the prevalence of individuals initially infected. Once individuals are infected, they then pass to the second part of the model (economic analytical model) as represented in Figure 1A and in Figure 1(main text).
